# Honeybee gut *Lactobacillus* modulates host learning and memory behaviors via regulating tryptophan metabolism

**DOI:** 10.1038/s41467-022-29760-0

**Published:** 2022-04-19

**Authors:** Zijing Zhang, Xiaohuan Mu, Qina Cao, Yao Shi, Xiaosong Hu, Hao Zheng

**Affiliations:** College of Food Science and Nutritional Engineering, China Agricultural University, 100083 Beijing, China

**Keywords:** Entomology, Symbiosis

## Abstract

Honeybees are highly social insects with a rich behavioral repertoire and are a versatile model for neurobiological research. Their gut microbiota comprises a limited number of host-restricted bacterial phylotypes that are important for honeybee health. However, it remains unclear how specific gut members affect honeybee behaviors. Here, we find that antibiotic exposure disturbs the gut community and influences honeybee phenotypes under field conditions. Using laboratory-generated gnotobiotic bees, we show that a normal gut microbiota is required for olfactory learning and memory abilities. Brain transcriptomic profiling reveals distinct brain gene expression patterns between microbiota-free and conventional bees. Subsequent metabolomic analyses of both hemolymph and gut samples show that the microbiota mainly regulates tryptophan metabolism. Our results indicate that host-specific *Lactobacillus* strains promote memory behavior by transforming tryptophan to indole derivatives that activate the host aryl hydrocarbon receptor. Our findings highlight the contributions of specific gut members to honeybee neurological processes, thus providing a promising model to understand host-microbe interactions.

## Abstract (web summary)

Eusocial honeybee is a versatile model for neurobiological research. Here, the authors established a link between honeybee gut *Lactobacillus* in bee learning and memory ability via indole derivatives that activate host’s aryl hydrocarbon receptor.

## Introduction

The gut microbiota plays a significant role in modulating host development and physiology, including metabolism and immune functions. Recent studies have shown the effects of symbiotic microbes on the central nervous system (CNS) and behavioral processes in humans and several animal systems. Microbes can impact the host brain through various pathways, such as immune modulation, and via microbial metabolites implicated in regulating the gut-brain axis^1^. Although it is unclear whether the neurotransmitters produced by certain gut bacteria (e.g., GABA, serotonin, dopamine) can reach the brain, considering the presence of the blood–brain barrier, the gut microbiota can influence brain physiology indirectly. For example, various short-chain fatty acids derived from microbial fermentation were suggested to regulate the rate-limiting enzymes involved in neurotransmitter biosynthesis^2^. Specifically, it has been documented that the gut microbiota modulates tryptophan (Trp) metabolism and that the produced serotonin, kynurenine (Kyn), and indolic compounds profoundly affect gut-brain interactions^3^. Although the functional connection between the microbiota and neurophysiology has been widely recognized, most recent studies have focused on mammalian and nonsocial-insect models. It is challenging to elucidate the contribution of individual gut members in mammals, which is partly due to the complex and unpredictable compositions of the gut community and the difficulty of maintaining and manipulating gnotobiotic animals^4^. Thus, models exhibiting high sociality and a specialized gut community would be ideal to fully understand the relationship between the gut microbiota and host social behaviors.

The honeybee (*Apis mellifera*) is an important agricultural pollinator for wild plants and crops, and studies have focused on bee health to prevent colony losses. In addition, the honeybee is a eusocial insect with distinct behavioral structures characterized by a complex range of interactive behaviors. It has been widely used as a model of perception, cognition, and social behaviors^5^. Honeybees have a simple and host-specialized gut microbiota, with 8–10 bacterial genera constituting over 97% of the community^6^. Most bacterial genera include closely related species with high strain-level diversity. Typically, two genera of lactic acid bacteria, *Lactobacillus* Firm5 and Firm4 (*Bombilactobacillus*^7^), are the most abundant, followed by *Gilliamella*, *Snodgrassella*, and *Bifidobacterium*. Bee gut bacteria inhabit diverse nutritional niches, play specific roles in the bee gut, and are beneficial for host nutrition, immune homeostasis, and pathogen resistance^8^. Although the impact of the gut community on honeybee health is relatively clear, few studies have searched for potential links between the gut microbiota and honeybee behaviors. Recent studies showed that gnotobiotic honeybees with a conventional (CV) gut microbiota had higher sugar sensitivity than microbiota-free (MF) bees^9^. Accordingly, the expression of genes associated with insulin/insulin-like signaling was also increased. Monocolonization with *Bifidobacterium asteroides*, the key polysaccharide degrader in the bee gut, elevates the concentration of juvenile hormone III derivatives in the gut, which may regulate host development^10^. These findings strongly suggest that the honeybee gut microbiota may contribute to host brain physiology and behavioral phenotypes. A recent study showed that oral supplementation with bee gut *Lactobacillus* increased the level of glycerophospholipids in the hemolymph and promoted the memory of bumblebees^11^. However, the contributions of particular gut members to honeybee behaviors via the regulation of metabolism remain unclear.

Herein, we investigated the role of the gut microbiota in altering honeybee behavioral phenotypes in the field. Our experiments showed that a conventional gut microbiota was needed for learning and memory abilities. Metabolic analyses of both gut and hemolymph samples suggested the effect of gut symbionts on host Trp metabolism. By generating monocolonized bees with symbiont isolates, we confirmed that bee gut *Lactobacillus* strains with aromatic amino acid aminotransferase (ArAT) convert Trp to an indolic aryl hydrocarbon receptor (AhR) agonist and improve learning and memory behaviors in an AhR-dependent manner.

## Results

### Antibiotic treatment disturbs honeybee phenotypes in hives

**Fig. 1:**
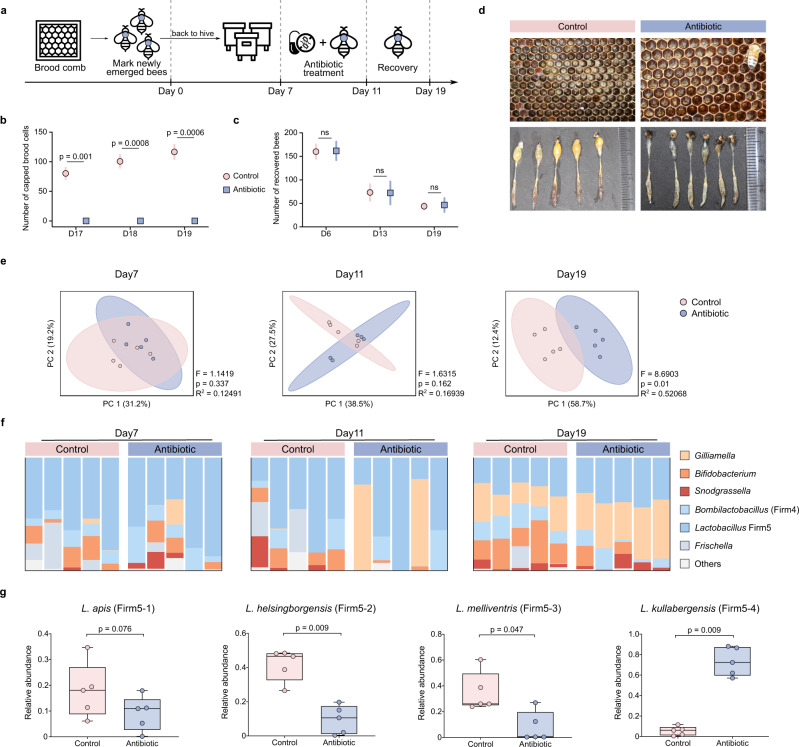
Antibiotic treatment affects the phenotypes of honeybees under field conditions. **a** Schematic of field experiments. Age-controlled bees were treated with tetracycline for 5 days (Days 7–11) in the hive and recovered for 7 days (Days 11–19). **b** Number of capped brood cells during the recovery stage (Days 17, 18, and 19) in three independent colonies of control and antibiotic-treated group, respectively. **c** The number of labeled workers recovered from three colonies of each group on Days 6, 13, and 19. Differences between antibiotic-treated bees and the control group were tested by multiple two-tailed *t*-test with Benjamini-Hochberg correction in (**b**) and (**c**). **d** Images of brood frames and dissected guts of control and antibiotic-treated groups. **e** Principal coordinate analysis of Bray-Curtis dissimilarity of gut community compositions of control and antibiotic-treated bees. Group differences were tested by permutational multivariate ANOVA (PERMANOVA). **f** Relative abundance of genera in metagenomic samples from control and antibiotic-treated groups. **g** Relative abundance of the four *Lactobacillus* Firm5 species (*n* = 5 bees for both groups). Differences between control and antibiotic-treated groups were tested by two-sided Mann-Whitney *u* test. ns, not significant. Error bars represent min and max. Source data are provided as a Source Data file.

First, we explored whether perturbation of the gut microbiota disturbs honeybee phenotypes under field conditions. To generate a single-cohort colony, we labeled newly emerged bees with color tags and placed them in empty hives with laying queens. One week after establishment of the hives, three hives were treated with tetracycline in wild honey, and the other three hives (control group) were fed wild honey on the same treatment schedule (Fig. 1a). We counted the number of capped brood cells and assessed the posttreatment survival of adult bees by counting the number of remaining marked bees. Typically, honeybee eggs hatch into larvae within 3–4 days, and the larval stage lasts for 6 days. Then, the larvae undergo metamorphosis inside sealed cells for another ~12 days. We started to check the status of pupation 12 days after egg laying (Day 15; Fig. 1a). Although there were an increasing number of capped brood cells in the control hives, no single capped brood was observed in the treatment group on Days 17, 18, and 19 (Fig. 1b). The number of recovered adult bees was not significantly different between the control and treatment groups either before (Day 6) or after (Day 13 and 19) antibiotic treatment (Fig. 1c). Developing eggs and larvae were present in the brood cells in control hives (Fig. 1d). However, only a few eggs were observed in the treatment group, and none of these hatched even after 19 days. Moreover, the rectums of bees from the control groups were full of yellow pollen, while those of treated bees were more translucent. This suggested a lack of pollen in the gut of treated bees, which could be evidence of bee malnutrition. Altogether, these results indicated that antibiotic-treated bees were less capable of rearing broods, while survivorship was not affected by tetracycline exposure during the experiment.

We further characterized the composition of the gut community at both the genus and species levels through metagenomic sequencing. Although the gut community composition displayed no significant difference at pretreatment sampling points, significant changes were observed after antibiotic exposure (Day 11; Fig. 1e). After recovery for 7 days (Day 19), the gut compositions were even more divergent. The treatment group had a higher fraction of *Gilliamella*, while the relative abundance of *Bifidobacterium* was reduced. Moreover, tetracycline treatment affected the genus- and species-level compositions of all core gut members (Fig. 1f, Supplementary Fig. 1). Specifically, the relative abundances of the four species of *Lactobacillus* Firm5 were most strongly affected by antibiotic exposure. The relative abundances of *Lactobacillus apis*, *Lactobacillus helsingborgensis*, and *Lactobacillus melliventris* were reduced in the antibiotic treatment group, while that of *Lactobacillus kullabergensis* was increased (Fig. 1g, Supplementary Fig. 1d).

### The gut microbiota influences honeybee learning and memory behaviors

**Fig. 2:**
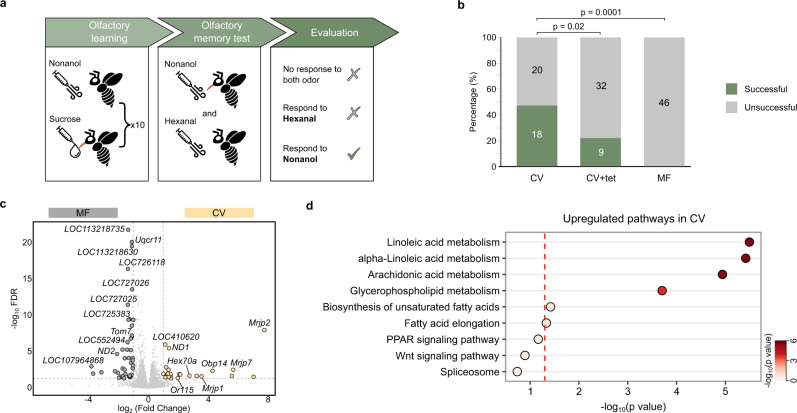
Gut microbiota alters honeybee learning and memory behaviors and the brain gene expression profile. **a** Olfactory learning and memory test design. 7-day-old conventionalized (CV), tetracycline-treated (CV + tet), and microbiota-free (MF) bees were tested. Bees responded to only the nonanol odor were considered successful. **b** Ratio of bees successful in the memory test. Group differences among CV (*n* = 38), CV + tet (*n* = 41), and MF (*n* = 46) bees were tested by Chi-squared test. **c** A volcano plot showing the differentially regulated genes (FDR < 0.05 and |log2FoldChange | > 1, Benjamini-Hochberg FDR method). **d** KEGG pathways upregulated in the brains of CV bees based on the differentially expressed genes (Fisher’s exact test). Source data are provided as a Source Data file.

The ability to discriminate and remember odors is critical for honeybee social behaviors, such as labor division, feeding organization, kin recognition, and mating^12^. Since we observed altered hive phenotypes under field conditions, we then examined whether gut microbiota colonization affects the olfactory learning and memory abilities of bees in the laboratory. Each individual of conventional (CV), tetracycline treated (CV + tet), and microbiota-free (MF) bee groups generated in the laboratory was trained for ten trials to associate the stimulus odor (nonanol) to a sucrose reward^13,14^. Although the honeybees learned the nonanol odor, the efficiency differed among groups, and the learning rate was higher for the CV bees (Supplementary Fig. 2b). The memory test was performed 3 h after associative learning, and bees that responded to only the nonanol odor were considered successful (Fig. 2a, Supplementary Movie 1). Almost 50% of the CV bees could remember the nonanol odor and distinguish the conditioned stimulus from the negative control odor (hexanol) (Fig. 2b). This percentage is similar to that for hive bees performing an olfactory learning task in a previous test^14^. In contrast, the proportion of individuals that remembered the odor was significantly lower in the antibiotic treatment group. Surprisingly, no MF bee exhibited successful memory behavior, suggesting that the gut microbiota can affect honeybee learning and memory abilities.

In-depth proteomic profiling of the brains of honeybees from the MF and CV groups identified a total of 3,427 proteins, 2,845 of which were detected in both the MF and CV groups (Supplementary Fig. 2c, Supplementary Data 2). Interestingly, enrichment analysis of proteins unique to the CV brain identified GO terms related to synaptic neurotransmission and transmembrane transport of cations/ions (Supplementary Fig. 2d), which are essential for fundamental functions in the honeybee CNS^15^. Notably, the muscarinic acetylcholine receptor (mAChR) involved in the cholinergic neurotransmitter system that processes olfactory signals^16^ was upregulated in the CV bees (Supplementary Fig. 2e). A splicing factor, U2af28, was upregulated in CV brains, implying altered splicing of genes in the brain.

### The gut microbiota regulates the expression of brain genes involved in honeybee learning and memory

Since the olfactory learning and memory behaviors of honeybees are primarily associated with the gene expression profile in the brain^17^, we explored the changes in the transcriptome induced by the gut microbiota. In total, RNA sequencing analysis revealed that 68 genes were differentially expressed in the CV bees compared to MF bees (|Log_2_ Fold Change | > 1, FDR < 0.05, Supplementary Data 3), and the two groups exhibited distinct brain gene expression profiles (Supplementary Fig. 3a). We found that the odorant-binding protein *Obp14* and olfactory receptor *Or115*, which are essential for the detection and identification of specific odors^18,19^, were upregulated in the CV group (Fig. 2c and Supplementary Fig. 3b). Three major royal jelly protein (MRJP) family genes (*mrjp 1*, *mrjp 2*, *mrjp 7*) and the hexamerin HEX70a involved in bee caste determination^20,21^ were also upregulated. Enrichment analysis of the differentially expressed genes showed that KEGG pathways, including the linoleic, alpha-linolenic, arachidonic acid, and glycerophospholipid metabolic pathways, were upregulated in the brains of microbiota-colonized bees (Fig. 2d).

### The gut microbiota mainly affects tryptophan metabolism

**Fig. 3:**
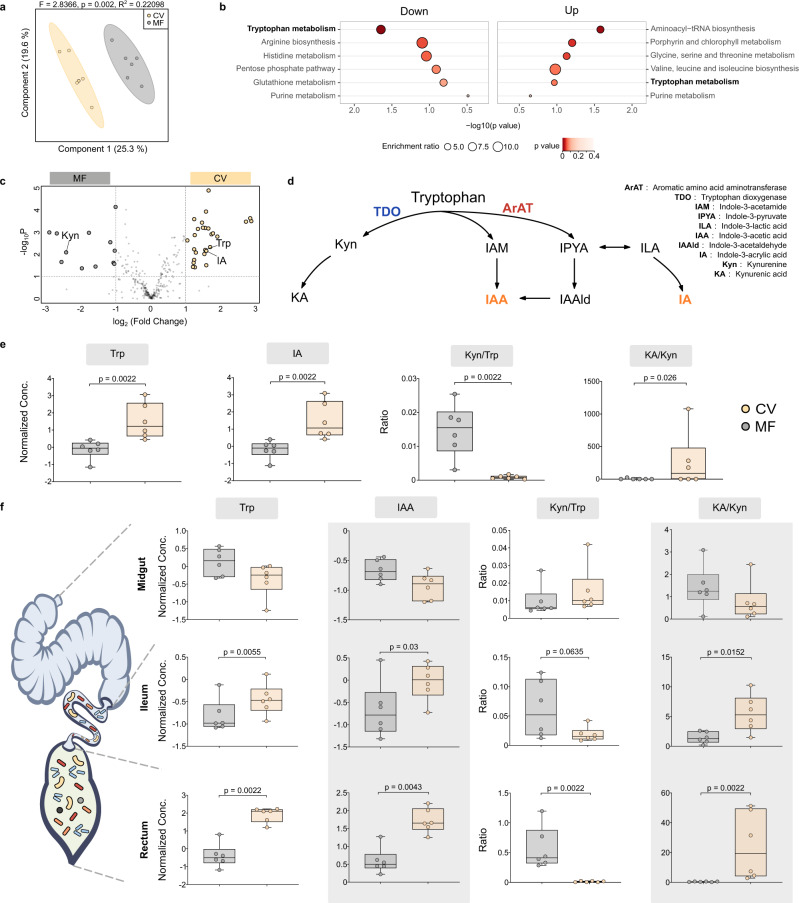
Tryptophan metabolism pathways in the gut and hemolymph are influenced by gut microbiota. **a** Sparse PLS-DA based on all metabolites detected in the hemolymph. Group differences were tested by permutational multivariate ANOVA (PERMANOVA). **b** The most enriched KEGG pathways down- and upregulated in the hemolymph of CV bees compared to MF group based on the differentially regulated metabolites (Fisher’s exact test). **c** A volcano plot showing the differentially regulated metabolites. **d** Key metabolites and enzymes of tryptophan metabolism via the kynurenine (Kyn) and indole pathways. Genes encoded by the host are shown in blue. Genes encoded by the gut bacteria are shown in red. Indole derivatives acting as AhR ligands are shown in orange. **e** Normalized concentration of tryptophan (Trp) and indole-3-acrylic acid (IA) in the hemolymph of MF and CV bees (*n* = 6 bees for both groups). The ratio of Kyn/Trp and kynurenic acid (KA)/Kyn was calculated (*n* = 6 bees for both groups). **f** Normalized concentration of Trp and indole-3-acetic acid (IAA), the Kyn/Trp ratio, and the KA/Kyn ratio in the midgut, ileum, and rectum of MF and CV bees (*n* = 6 bees for both groups). Group differences were tested by two-sided Mann-Whitney *u* test. Error bars represent min and max. Source data are provided as a Source Data file.

To determine the key metabolites regulated by gut symbionts potentially impacting the circulatory system^2^, we performed quasi-targeted metabolomic analysis of honeybee hemolymph samples. In total, 326 metabolites were identified in the MF and CV bees (Supplementary Data 4), and the metabolic signatures of the hemolymph samples were significantly different between the two groups (Fig. 3a). Interestingly, KEGG pathway enrichment analysis highlighted that metabolites involved in amino acid metabolic pathways, such as tryptophan, serine, and valine metabolism, were regulated by the gut microbiota. Notably, the tryptophan metabolism pathway was either up- or downregulated by gut microbes. Thus, we wished to identify the metabolites belonging to this pathway that were altered. A volcano plot showed that the levels of both tryptophan and indole-3-acrylic acid (IA) were elevated in the CV bee hemolymph, while the level of Kyn was increased, when the gut microbiota was depleted (Fig. 3c).

Dietary Trp can be catabolized by gut bacteria into a variety of indole derivatives, such as IA and indole-3-acetic acid (IAA), which are key components for intestinal homeostasis^22^ (Fig. 3d). Alternatively, Trp is metabolized through the Kyn pathway mediated by host enzymes, which leads to the production of Kyn and kynurenic acid (KA). The CV bees had higher levels of Trp and IA in the hemolymph (Fig. 3e). Furthermore, the Kyn/Trp ratio (a biomarker of the activity of the Kyn pathway^23^) was decreased, while for the KA/Kyn^23^ ratio was increased, in the presence of the gut microbiota. These results indicated that the gut microbiota suppressed Trp metabolism through the Kyn pathway, which has been widely associated with neurodegenerative diseases^24^. It has been documented that the gut microbiota mostly affects amino acid metabolism pathways in the gut. We revisited our metabolomic data obtained from different gut compartments of the CV and MF bees^9^. Although IA was not detected with our previous metabolomic approach, the levels of both Trp and IAA were increased in the ileum and rectum (Fig. 3f), where most gut bacteria reside. Accordingly, the Kyn/Trp and KA/Kyn ratios were also regulated, as observed in the hemolymph samples. Interestingly, none of these indices were altered between the CV and MF bees within the midgut, which is colonized by few microbes. Similarly, antibiotic treatment under field conditions inhibited the microbial indole pathway and shifted Trp metabolism toward the production of Kyn in the gut (Supplementary Fig. 4, Supplementary Data 5). Altogether, these results indicated that the honeybee gut microbiota mostly influenced Trp metabolism by transforming Trp to indole derivatives and limiting the host Kyn pathway.

### *Lactobacillus* Firm5 improves learning and memory behaviors via the indole-AhR signaling pathway

**Fig. 4:**
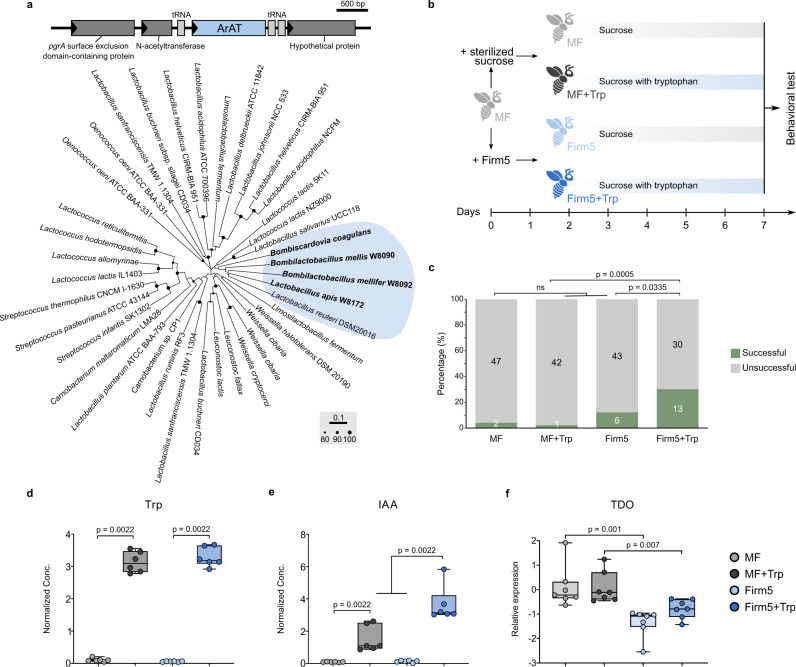
*Lactobacillus apis* with ArAT alters tryptophan metabolism and improves honeybee memory retention. **a** Graphical representation of the gene locus encoding ArAT (locus_tag: H3R21_07190). Neighbor-joining tree based on the amino acid sequences of ArAT. Sequences from honeybee *Lactobacillus* spp. and *Bombiscardovia* are clustered together with that of *Lactobacillus reuteri* from human gut. Nodes with high bootstrap values are marked (> 80%; 1000 replicates). **b** Experimental design: MF bees were fed with 50% sucrose with (MF + Trp) or without 12% tryptophan (MF). MF bees colonized with *L. apis* were provided with 50% sucrose with (Firm5 + Trp) or without 12% tryptophan (Firm5). Each group contains 50 bees. The learning and memory performance was tested at 7 days of age. **c** Ratio of bees successful in the memory test. Group differences among MF (*n* = 49), MF + Trp (*n* = 43), Firm5 (*n* = 49), a*n*d Firm5+Trp (*n* = 43) bees was tested by Chi-squared test. ns, not significant. **d**, **e** Boxplots of the normalized concentration of (**d**) Trp and (**e**) IAA in th**e** gut of MF, MF + Trp, Firm5, and Firm5+Trp bees (*n* = 6 bees for all groups). **f** Relative expressions of the TDO gene in the gut of MF, MF + Trp, Firm5, and Firm5+Trp bees (*n* = 7 bees for all groups). Group differences of metabolite concentrations and gene expression levels were tested by two-sided Mann-Whitney *u* test. Error bars represent min and max. Source data are provided as a Source Data file.

Indole derivatives are generated via the indole pathway by many human intestinal anaerobic bacteria, which encode functional ArATs (Fig. 3d). ArAT is a key enzyme that is conserved in many bacterial species, but not all of these species can convert Trp to aroma compounds^25^. We identified the honeybee gut species that possessed potential active ArATs in their genomes using BLAST. Interestingly, we found that only strains of *Lactobacillus* Firm5 and *Bombilactobacillus* and one strain of *Bombiscardovia coagulans* (family *Bifidobacteriaceae*) had ArAT subfamily I genes^26^. Phylogenetic analysis showed that sequences from bee gut bacteria clustered together with the sequence from *Lactobacillus reuteri* (Fig. 4a). Notably, these sequences were distantly related to the sequence from *Lactobacillus johnsonii*, which could not produce indole derivatives in ex vivo stomach cultures^25^.

We then examined whether *L. apis* converts Trp to indole derivatives and subsequently promotes honeybee learning and memory behaviors. First, we generated gnotobiotic bees monocolonized with *L. apis* strain W8172 (Firm5) encoding an ArAT closely related to that of *L. reuteri* (Fig. 4a, b). To eliminate the effect of dietary amino acids, we fed bees with only sucrose syrup, not pollen grains, in this experiment (Fig. 4b). Although the olfactory learning performance was not different among groups (Supplementary Fig. 5b), bees monocolonized with *L. apis* and supplemented with dietary Trp performed significantly better in the memory test (Fig. 4c). However, without the addition of Trp, *L. apis* colonization alone was not sufficient to promote memory behavior. Thus, *L. apis* promoted honeybee behaviors only during the generation of indole derivatives under high levels of Trp. Indeed, the gut concentration of Trp in both groups subjected to dietary Trp supplementation was significantly higher (“MF + Trp” and “Firm5+Trp”; Fig. 4d). However, IAA was mostly elevated in the gut of members of the Firm5+Trp group (Fig. 4e). In addition, the levels of the precursors of both IAA (IAAld) and IA (ILA) were increased in Firm5-colonized bees fed dietary Trp (Supplementary Fig. 5c, d). These results confirm that *L. apis* can generate indole derivatives from dietary Trp in the gut.

Trp metabolism along the Kyn pathway is initiated mainly by the host enzymes indoleamine-2,3-dioxygenase (IDO) and Trp 2,3-dioxygenase (TDO) in humans^3^. In honeybees, TDO is the sole enzyme that catalyzes the initial step of the Kyn pathway, as also found in *Drosophila*^24^. The expression of TDO was inhibited in the Firm5-colonized bees with or without the addition of Trp to the diet compared to the expression in the MF groups (Fig. 4f). This is consistent with the altered Kyn/Trp and KA/Kyn ratios found in the hemolymph and gut samples (Fig. 3), which indicated that the host Kyn pathway was suppressed by the gut microbiota.

**Fig. 5:**
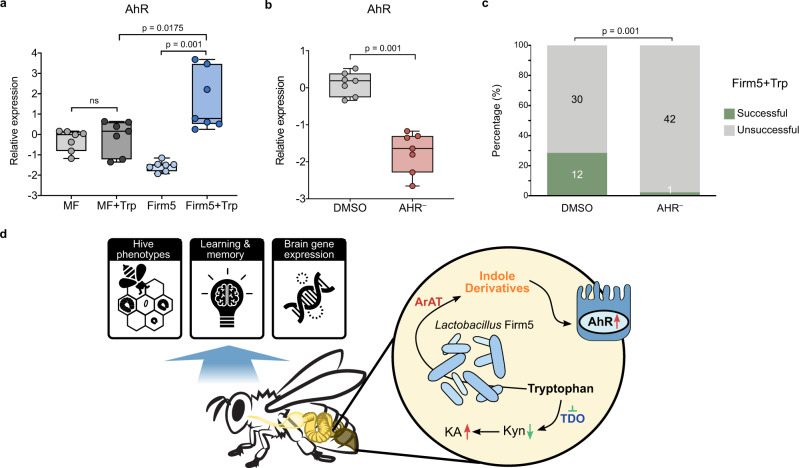
*Lactobacillus apis* promotes learning and memory behaviors in an AhR-dependent manner. **a** Relative expressions of the AhR gene in the gut of MF, MF + Trp, Firm5, and Firm5+Trp bees (*n* = 7 bees for all groups). **b** Relative expressions of the AhR gene in the gut of Firm5+Trp bees treated with AhR antagonist (AhR^–^) dissolved in DMSO (*n* = 7 bees for both groups). Group differences of gene expression levels were tested by two-sided Mann-Whitney *u* test. Error bars represent min and max. **c** Ratio of bees successful in the memory test. Group difference between the DMSO (n = 42) and AHR^–^ (*n* = 43) was tested by Chi-squared test. **d** Graphical summary of the effects of gut microbiota on honeybee behavioral phenotypes. Gut bacteria encoding ArAT produce indolic AhR ligands and suppress the host TDO in the Kyn pathway. An altered gut microbiota significantly impacts the hive phenotypes under field conditions, learning and memory abilities, and gene expression patterns in the brain. Source data are provided as a Source Data file.

Indole derivatives produced by gut bacteria are ligands of the aryl hydrocarbon receptor (AhR), a ligand-activated transcription factor with important physiological roles. Since gut bacteria mainly affect the levels of indolic compounds that are AhR agonists, we hypothesized that gut bacteria promote honeybee behaviors via the activation of AhR in the gut. Our results showed that the expression of AhR in the gut was stimulated only upon colonization by *L. apis* with the addition of Trp in the diet (Fig. 5a), indicating that AhR was activated by the indolic ligands produced by gut bacteria. Then, we explored whether the gut bacteria-promoted behaviors were dependent on AhR. When we treated bees with an AhR antagonist, AhR expression was successfully suppressed in gut epithelial cells (Fig. 5b). We retested the learning and memory behaviors of the Firm5+Trp group of bees treated with the AhR antagonist. *L. apis* failed to promote honeybee learning (Supplementary Fig. 5e) and memory (Fig. 5c) behaviors when AhR was inhibited. This indicated that the gut bacteria improved honeybee behaviors by producing indole derivatives that modulate AhR activation.

## Discussion

In this study, we showed that bees with disturbed gut communities impaired hive phenotypes under field conditions, and laboratory experiments using gnotobiotic honeybees showed the effects of the gut microbiota on host learning and memory behaviors with shifts in Trp metabolism. Monocolonization by *Lactobacillus* strains with ArAT can regulate Trp metabolism, increase the levels of indolic AhR ligands, suppress TDO activity in the Kyn pathway, and improve honeybee learning and memory performance. Moreover, inhibition of AhR resulted in impaired learning and memory abilities, highlighting the mechanisms implicated in host-microbiota interactions in honeybees (Fig. 5d).

Honeybees are eusocial insects that exhibit complex social, communication, and navigational behaviors with rich cognitive repertoires, such as color vision, pattern recognition, learning and memory^27^. Within a colony, honeybees are characterized by labor division, showing striking behavioral and physiological differences between castes^28^. Although the gut microbiota composition is conserved in worker bees, it differs among individuals with different behaviors and physiologies, such as individuals with of different castes, ages, and worker tasks^29^, which suggests that the gut microbiota might be involved in the behavior of honeybees. While our previous study showed that the bee gut microbiota alters olfactory sensitivity^9^, the impact of the microbiota on additional behavioral symptoms has not been described. Olfaction and learning and memory abilities play a crucial role in honeybees for coping with individual and social tasks, such as feeding and foraging^30^. Social isolation and group size can affect honeybee learning, while memory is not related to group size^31^. Thus, we used the same group size for different treatment groups (20 bees per cup cage) to evaluate the impact of gut bacteria. Our results indicated that a conventional gut microbiota is required for learning and memory formation in honeybees.

Notably, we performed only the associative appetitive learning assay in this study, and the roles of the gut microbiota in other bee behaviors are not clear. For example, appetitive and aversive learning behaviors are mediated by relatively independent neural systems in honeybees^32^. A recent study found that oral supplementation with bee gut *Lactobacillus* Firm5 altered the long-term memory of bumblebees. Interestingly, the abundance of glycerophospholipids increased in the hemolymph^11^. While our results indicated that honeybee gut *Lactobacillus* enhanced host memory, the gut bacteria did not change lipid metabolism in either gut or hemolymph samples. These findings suggest that the gut microbiota may regulate host behaviors via distinct pathways. However, Leger and McFrederick^33^ found that the effect of gut bacteria on the visual learning and memory behaviors of bumblebees is not obvious, which further suggests that the mechanism underpinning gut-brain interactions differs for social bees or is distinct for olfactory and visual processing.

Our RNA-seq analysis of gnotobiotic bee brains showed that the expression of several genes related to honeybee learning and memory behaviors was altered by gut bacteria. For example, genes encoding MRJPs are important for the learning abilities of honeybees. Specifically, MRJP1 is synthesized in the hypopharyngeal gland, and its expression level is always reduced in the mushroom bodies of workers with poor learning performance, suggesting its involvement in the development of learning ability in honeybees^34^. Consistent with this, the expression levels of *mrjp1* and *mrjp4* were repressed in the brains of imidacloprid-treated bees exhibiting impaired learning ability^17^. We found that many MRJP genes were upregulated in the CV bees, suggesting that gut bacteria regulate brain gene expression and promote bee learning and memory.

Our hive experiments demonstrated that perturbation of the gut microbiota disturbed bee phenotypes in the field. The number of capped brood cells is a measure of colony strength, which could also be influenced by the status of the egg-laying queen and the colony population size^35^. However, the total number of individual bees was not obviously reduced, and newly laid eggs were continuously observed in the treatment hives, implying that the perturbation of the gut microbiota affects colony reproduction. However, it is difficult to separate the effects of antibiotics on the gut microbiome from the effects on the host itself. It was shown that antibiotics perturbed the fine-scale genetic diversity of the gut community^36^ and increased the susceptibility to opportunistic pathogens, which resulted in a reduced survival rate of bees in the hive^37^. In addition, tetracycline treatment during adult life can delayed the behavioral development from nurses to foragers^38^. Moreover, tetracycline has been shown to have a variety of effects on reproductive fitness and endocrine signaling in both vertebrate and invertebrate models, such as *Drosophila*^39^, zebrafish^40^, and rats^41^. Thus, we could not exclude the possibility that the observed effects of antibiotic treatment might be caused by their direct impact on the hosts.

It was recently shown that nestmate recognition cues are defined by gut bacteria, possibly via modulation of host metabolism or by the direct generation of colony-specific blends of cuticular hydrocarbons^42^. In leaf-cutting ants and termites, gut microbiota suppression by antibiotics also influences the recognition behavior toward nestmates, which may be directed by bacterial metabolites as recognition cues in the feces^43,44^. Nevertheless, the effect of the gut community is driven mainly by microbial metabolism, specifically the amino acid and lipid metabolic pathways, which can further influence the circulation system of the host^45,46^. Recently, gut Trp metabolism was found to be associated with neuropsychiatric disorders in humans and a *Drosophila* model, characterized by reduced plasma levels of Trp^47^ and high IDO1 activity^23^. We found that the levels of Trp and the indole derivatives were also elevated in the CV bee gut and hemolymph compared to their bacteria-free counterparts. Trp metabolism in the gut mainly involves the conversion of Trp into indole derivatives by resident bacteria and the production of Kyn mediated by host enzymes^48^. The indole pyruvate route is one of the main pathways for indolic compound synthesis. ArAT is a key enzyme converting Trp to indole derivatives conserved in many bacterial species, including lactobacilli from the human intestine^49^. The honeybee gut harbors two major groups of lactobacilli, *Bomilactobacillus* and *Lactobacillus*, which are two recently reclassified bacterial genera formerly known as *Lactobacillus* Firm4 and Firm5, respectively^7^. They are the most abundant gut members prevalent in both honeybees and bumblebees. We found that ArATs were present in the genomes of both *Bomilactobacillus* and *Lactobacillus*, and these were closely related to that from *L. reuteri*, which can produce aroma compounds in the human intestine^49^. Lactobacilli rely primarily on the availability of easily fermentable sugars and amino acids. *Lactobacillus* strains encode genes related to carbohydrate metabolism, suggesting that they are also essential fermenters in the honeybee gut. However, lactobacilli are also auxotrophic for amino acids and switch from sugar to Trp as a major energy source, catabolizing Trp to indole derivatives under carbohydrate-limiting conditions^50^. Indeed, our in vivo test showed that *L. apis* W8172 produced high levels of indole derivatives in the gut only with the administration of dietary Trp (Fig. 4e). Notably, not all strains of honeybee lactobacilli harbor ArAT genes. Multiple species have been defined within the bee *Lactobacillus* and *Bombilactobacillus* groups, and further strain-level variations have been repeatedly reported for honeybee gut species^51,52^. This suggests that the distinct nutritional adaptations of bee gut species may facilitate niche differentiation, allowing the coexistence of closely related members in the bee gut^51^.

Multiple indole derivatives have been identified as agonists of AhR, a ligand-activated transcription factor playing important roles in human health^53^. In both *Caenorhabditis elegans* and *Drosophila melanogaster*, indoles produced by the microbiota extend the host lifespan via AhR and induce a youthful gene expression profile^54^. Honeybees also possess a gene (LOC411264) encoding AhR, but its functions, such as the xenobiotic tolerance found in other insects^55^, are still not clear. We found that colonization by *L. apis* in the bee gut stimulated the expression of AhR in gut epithelial cells, but only with dietary supplementation with Trp. This indicates that the activation of AhR relies on the indole derivatives generated by gut bacteria. Moreover, *L. apis* could not improve host learning and memory behaviors when AhR was suppressed by the antagonist, which indicated that the effects of honeybee gut bacteria are at least partially mediated by host AhR signaling. The role of AhR in the regulation of the immune response has been extensively studied, while its participation in the gut-brain axis remains elusive^56^. Experiments using honeybees as a new model with a manipulated microbiota will help us reveal the underlying mechanisms, specifically the potential of AhR as a therapeutic target for human social pathologies^56^.

It has been shown that gut microorganisms may influence social behaviors across diverse animal hosts^57^. Honeybees are colonial and highly social organisms with multiple characteristic behaviors, offering an experimental tool to investigate the relationship between the microbiota and host brain functions and uncover the causal mechanisms underlying sociability. While our results indicated the critical role of specialized bee gut symbionts in the learning and memory ability implicated in other complex social behaviors^12^, further evaluation of the effect of the microbiota on different behaviors, such as kin recognition and social organization, would assist in fully understanding the mechanisms underlying gut-brain interactions. The development of genetic tools for manipulating both the bee host and gut bacteria would facilitate the investigation of the molecular basis of host-microbe interactions via the gut-brain axis.

## Methods

### Treatment of microbiota-free, conventional, and mono-colonized honeybees

Insects: honeybees (*Apis mellifera*) used in this study were from colonies maintained in the experimental apiary of the China Agricultural University. Pupae and newly emerged bees used in all the experiments were obtained from brood frames taken from the experimental hives and kept in an incubator at 35 °C, with humidity of 50%. There is no current requirement regarding insect care and use in research. Honeybees were cared for daily with adequate food during the experimental period. For tissue collection, bees were collected gently and immediately euthanized by CO_2_ anesthesia before dissection to reduce any unnecessary duress.

Microbiota-free (MF) bees were obtained as described by Zheng et al.^51^ with modifications (Supplementary Figs. 2a and 5a). Late-stage pupae were removed manually from brood frames and placed in sterile plastic bins. The pupae emerged in an incubator at 35 °C, with humidity of 50%. Newly emerged MF bees (Day 0) were kept in axenic cup cages with sterilized sucrose syrup (50%, wt/vol) for 24 h and divided into two groups: 1) MF and 2) conventional (CV) bees. For each setup, 20–25 MF bees (Day 1) were placed into one cup cage, and the bees were feeding on the corresponding solutions or suspensions for 24 h. For the MF group, 1 ml of 1×PBS was mixed with 1 ml of sterilized sucrose solution (50%, wt/vol) and 0.3 g sterilized pollen. For the CV group, 5 µl homogenates of freshly dissected hindguts of nurse bees from their hives of origin were mixed with 1 ml 1×PBS, 1 ml sterilized sucrose solution (50%, wt/vol) and 0.3 g sterilized pollen. Then MF and CV bees were provided sterilized sucrose (0.5 M) with sterile pollens and kept in an incubator (35 °C, RH 50%) until day 7.

To verify the effect of tryptophan metabolism on the host, newly emerged bees (Day 0) as described above were divided into four groups: 1) MF, 2) MF supplemented with tryptophan (MF + Trp), 3) *Lactobacillus* Firm5, and 4) Firm5+Trp bees. For each setup, 20–25 MF bees (Day 1) were placed into one cup cage, and the bees were feeding on the corresponding solutions or suspensions for 24 h. For the MF and MF + Trp groups, 1 ml of 1×PBS was mixed with 1 ml of sterilized sucrose solution (50%, wt/vol). For the Firm5 and Firm5+Trp groups, stock of *Lactobacillus apis* strain W8172 in 25% glycerol stock at −80 °C were resuspended in 1 ml 1×PBS (Solarbio, Beijing, China) at a final OD_600nm_ of 1, and then mixed with 1 ml sterilized sucrose solution (50%, wt/vol). Then MF and Firm5 bees were provided sterilized sucrose (0.5 M), while MF + Trp and Firm5+Trp bees were provided with sterilized sucrose (0.5 M) with 12 mg/ml tryptophan but without pollen grains^58^. All bees were kept in an incubator (35 °C, RH 50%) until day 7. All bees used here all came from the same colony. Bees coming from the same cup cage were considered as one replicate of each group.

### Bacterial load quantification

Colonization levels of MF and Firm5 bees were determined by colony-forming units from dissected guts, as described by Kwong et al.^59^. Colonization levels of CV bees were determined by quantitative PCR as previously described by Kešnerová et al.^10^. All qPCR reactions were carried out in a 96-well plate on the QuantStudio 1 Real-Time PCR system (Thermo Fisher Scientific, Waltham, MA, USA) with the thermal cycling conditions as follows: denaturation stage at 50 °C for 2 min followed by 95 °C for 2 min, 40 amplification cycles at 95 °C for 15 s, and 60 °C for 1 min. Melting curves were generated after each run (95 °C for 15 s, 60 °C for 20 s and increments of 0.3 °C until reaching 95 °C for 15 s) to compare dissociation characteristics of the PCR products obtained from gut samples and positive control. Universal bacteria primers (Forward: 5′-AGGATTAGATACCCTGGTAGTCC-3′, Reverse: 5′-YCGTACTCCCCAGGCGG-3′)^10^ and *Apis mellifera* actin (Forward: 5′-TGCCAACACTGTCCTTTCTG-3′, Reverse: 5′-AGAATTGACCCACCAATCCA-3′)^60^ were used here. Standards for target genes cloned into the pCE2 TA/Blunt-Zero Vector (Vazyme Biotech; Nanjing, China) were created by PCR amplification of the genomic DNA of *Gilliamella apicola* strain W8127. The copy number of plasmids was determined by performing standard curves on serial dilutions of plasmids containing the target sequence. The final concentrations of the plasmid in these template samples ranged from 10^1^–10^7^ copies per μl. Each reaction was performed in triplicates on the same plate in a total volume of 10 μl (0.2 μM of each forward and reverse primer; and ChamQ Universal SYBR qPCR Master Mix, Vazyme Biotech) with 1 μl of DNA. Each plate contained a positive control and a water control. The data was analyzed using the QuantStudio Design and Analysis Software (version 1.5.0; Thermo Fisher Scientific). After the calculation of the bacterial 16 S rRNA gene copies, normalization with the actin gene was carried out to reduce the effect of gut size variation and extraction efficiency. In brief, bacterial 16 S rRNA gene copies were normalized to the medium number of actin gene copies by dividing by the ‘raw’ copy number of actin for the given sample and multiplying by the median number of actin gene copies across all samples.

### Tissue collection

The whole guts were dissected by tweezers sterilized with 75% alcohol. Dissected guts were directly crushed in 25% (vol/vol) glycerol on ice for bacterial load quantification or collected into an empty 1.5-ml centrifuge tube for metagenomic sequencing and metabolomics analysis. All gut samples were frozen at −80 °C until analysis. Honeybee brains were collected using a dissecting microscope (Canon). Individual bee was fixed on beeswax using two insect needles through the thorax. After removing the head cuticle, the whole brain was placed on a glass slide and soaked in RNAlater (Thermo; Waltham, MA, USA) or proteinase inhibitor (Roche; Mannheim, Germany) for gene expression profiling, proteome analysis, and neurotransmitters concentration quantification. Then hypopharyngeal glands, salivary glands, three simple eyes, and two compound eyes were carefully removed. Dissected brains were kept frozen at −80 °C. Hemolymph was collected using a 10 μl pipettor (Eppendorf; Hamburg, Germany) from the incision above the median ocellus. A minimum of 50 μL of hemolymph was collected from10 bees into a 1.5-ml centrifuge tube. During the collection process, tubes are temporarily preserved on dry ice and subsequently stored at −80 °C until analysis.

### Learning and memory assay

We measured the olfactory learning and memory ability of seven-day-old MF, CV, CV + tet, and Firm5 bees. MF, CV, and Firm5 bees were generated as described above. CV + tet bees were fed 450 μg/ml (final concentration) of tetracycline suspended in sterilized 0.5 M sucrose syrup on Day 5 after the eclosion for 24 h and then were fed sucrose syrup for another 24 h for recovery. Experiments of olfactory learning and memory were performed as previously described^13,14^ with modifications (Fig. 2a). In brief, bees were starved for 2 h by removing sugar syrup and bee bread from the cup cage before the test. Then, they were mounted to a modified 0.8 mm wide bullet shell with sticky tape restraining harnesses (Supplementary Movie 1). The whole experiment was performed in a room with a stable light source at room temperature. Each bee individual was checked for their intact proboscis extension response (PER) by touching the antennae with 50% sucrose solution without subsequent feeding 15 min before the experiment. Bees that do not show PER to sucrose were removed for further experiments. Nonanol (olfactory learning; Sigma-Aldrich; Saint Louis, MO, USA) and hexanal (negative control; Macklin; Shanghai, China), which could be distinguished by honeybee, were used as odor sources. The odor was produced by pricking holes on a 0.8 cm wide filter paper and soaking it in 0.5 ml nonanol or hexanal, and the filter paper was then slipped into a 10 ml injector. During conditioning, a harnessed bee was placed in front of an exhaust fan to prevent odor build-up in subsequent experiments. Bees were trained for 10 trials with an inter-trial interval of 10 min to associate nonanol odor as the conditioned stimulus with a reward of 50% sucrose solution as the unconditioned stimulus.

At the beginning of each trial, the harnessed bee was placed inside the arena for 5 seconds to familiarize the experimental context. After that, the nonanol odor was presented before its antennal for 6 sec. A 0.4 µl droplet of sucrose solution was then delivered to the bee using a syringe needle, which directly touched the proboscis to evoke PER. Any bee that responded with a conditioned response on the first trial was removed from the experiment during the experiment. Once the 10 trials of a conditioning session were completed, bees were kept in the dark without being fed for 3 h. Two unreinforced olfactory memory tests were administered 3 h after olfactory conditioning: one with the conditioned stimulus odor (nonanol) and one with a novel odor (hexanal). Bees in a low physical condition or dying were removed for further test. The order of presentation was randomized across subjects. A clean and tasteless injector was delivered to the bee after each odor test to exclude visual memory of reward during olfactory conditioning. Bees extending the proboscis only to nonanol odor were considered as successful individuals (Fig. 2a). After the last odor stimulation performed in the memory test, PER was tested in all bees by applying 50% sucrose solution to the antennae. Bees that do not show PER to sucrose were discarded from the data.

### Hive experiment

The fieldwork took place in 2019 at the apiary of China Agricultural University, Beijing, China, and the experiment was performed twice in July and August, respectively. To observe the effect of gut microbiota on the hive phenotypes with the same age, two treatment groups each containing three independent single-cohort colonies for were set up as previously described^61^. Briefly, brood frames were collected from a single hive, and adult bees were brushed off. The frames were then kept in the laboratory incubating at 35 °C and 50% relative humidity. In two days, about 1,000 bees emerged from each frame in the incubator, and we labeled 300 individuals with colored tags on their thorax. All newly emerged bees were then introduced to new empty hives together with a newly mated laying queen^62^. Two hives for control and treatment were established. Control colony bees were fed wild honey along with the whole experiment, and treatment groups were fed wild honey suspended with 450 μg/ml of tetracycline (final concentration) from Day 7 after the establishment of hives (Fig. 1a). The antibiotic treatment lasted for 5 days. The number of capped brood cells was counted every day, and post-treatment survival in the hive was assessed by counting the number of remaining marked bees of the whole hive^37^. Marked bees for both control and treatment groups were collected from each hive at time points of Day 7, 11, and 19 following the setup of hives, and the hind guts and brain tissue were dissected. All samples were stored at −80 °C until analysis.

### Topical treatment of AhR antagonist

The topical treatment on the abdomen with AhR antagonist was performed as previously described^63,64^ with modifications. The AhR antagonist CH223191 (Sigma-Aldrich) was resuspended in dimethyl sulfoxide (DMSO; Sigma-Aldrich) at a concentration of 1ug/µl. For the AHR^−^ group, 1 µl of this solution was applied to the center of the dorsal abdomen (segment IV) of Firm5+Trp bees (Day 7). Firm5+Trp bees (Day 7) treated with 1 µl of DMSO on the center of the dorsal abdomen (segment IV) served as control (DMSO group). Bees were held immobile for 30 s after treatment to allow the solvent to penetrate the cuticle. Treated bees were kept in an incubator (35 °C, RH 50%) for 24 h. Then the learning and memory ability of treated bees were measured as described above. After behavior test, gut samples were collected for further analysis.

### RNA extraction and quantitative PCR

Each dissected gut was homogenized with a plastic pestle, and total RNA was extracted from individual samples using the RNA-easy Isolation Reagent (Vazyme) according to the manufacturer’s protocols. cDNA then was synthesized using the HiScript III RT SuperMix for qPCR (Vazyme). qPCR was performed using the ChamQ Universal SYBR qPCR Master Mix (Vazyme) and QuantStudio 1 Real-Time PCR Instrument (Thermo Fisher Scientific, Waltham, MA, USA) in a standard 96-well block (20-µl reactions; incubation at 95 °C for 3 min, 40 cycles of denaturation at 95 °C for 10 s, annealing/extension at 60 °C for 20 s). The primers for gene AhR (LOC411264; Forward: 5′-AGCGTGATACTTGGAGTGGC-3′, Reverse: 5′-ACGTCGATTACCCGCCAAAT-3′), TDO (LOC410828; Forward: 5′-TCGATTTTTCATCAATAGTGACAGG-3′, Reverse: 5′-CCGAATTCCAACCATTGCAGG-3′) were used here. The *A. mellifera actin* gene was chosen as the control, and relative expression was analyzed using the 2^-∆∆CT^ method.

### Gut DNA extraction and metagenomic sequencing

Bee individuals of either control or antibiotic groups were sampled on Day 7, 11, and 19 during the hive experiment (Fig. 1a). Total genomic DNA of the gut microbiota was extracted from the whole gut homogenate using the CTAB-based method as previously described^9^. DNA samples were sent to Novogene Bioinformatics Technology Co. Ltd. (Beijing, China) for shotgun metagenome sequencing. Sequencing libraries were generated using NEBNext Ultra^TM^ II DNA Library Prep Kit for Illumina (New England Biolabs; Ipswich, MA, USA), and the library quality was assessed on Qubit 3.0 Fluorometer (Life Technologies; Grand Island, NY, USA) and Agilent 4200 (Agilent, Santa Clara, CA) system. The libraries were then sequenced on the Illumina Novaseq 6000 platform (Illumina; San Diego, CA, USA), and 150 bp paired-end reads were generated. The genus- and species-level community structure of each metagenomic sample was profiled following the Metagenomic Intra-Species Diversity Analysis System (MIDAS) pipeline^65^. A custom bee gut bacteria genomic database was generated based on 407 bacterial isolates from honeybees and bumblebees (Supplementary Data 1). Before the classification, we removed reads belonging to the honeybee reference genome (version Amel_HAv3.1) using KneadData v 0.7.3. We then ran the ‘species’ module of the ‘run_midas.py’ and ‘merge_midas.py’ scripts in MIDAS with our custom bacterial genome database, which aligned reads to universal single-copy gene families of phylogenetic marker genes using HS-BLASTN to estimate the abundance of genus and species for each sample. Local alignments covering <70% of the read or failing to satisfy the gene-specific species-level percent identity cut-offs were discarded.

### Brain gene expression analysis

Total RNA was extracted from individual brains using the Quick-RNA MiniPrep kit (Zymo; Irvine, CA, USA). RNA degradation and contamination were monitored on 1% agarose gels, and the purity was checked with the NanoPhotometer spectrophotometer (IMPLEN; CA, USA). RNA integrity was assessed using the RNA Nano 6000 Assay Kit of the Bioanalyzer 2100 system (Agilent Technologies; Santa Clara, CA, USA). RNA sequencing libraries were generated using NEBNext Ultra RNA Library Prep Kit for Illumina (New England BioLabs; Ipswich, MA, USA), and index codes were added to attribute sequences to each sample. The clustering of the index-coded samples was performed on a cBot Cluster Generation System using TruSeq PE Cluster Kit v3-cBot-HS (Illumina; San Diego, CA, USA), and the library preparations were then sequenced on an Illumina NovaSeq 6000 platform (Illumina; San Diego, CA, USA) and 150 bp paired-end reads were generated. The sequencing quality of individual samples was assessed using FastQC v0.11.5 with default parameters. An index of the bee reference genome (Amel_HAv3.1) was built using HISAT2 v2.0.5^66^, and the FastQC trimmed reads were then aligned to the built index using HISAT2 v2.1.0 with default parameters. Gene expression was quantified using HTSeq v0.7.2^67^ with mode ‘union’, only reads mapping unambiguously to a single gene are counted. In contrast, reads aligned to multiple positions or overlapping with more than one gene are discarded. If it were counted for both genes, the extra reads from the differentially expressed gene may cause the other gene to be wrongly called differentially expressed, so we chose ‘union’ mode.

Differential gene expression analysis was performed using the DESeq2 package^68^ in R. We modeled read counts following a negative binomial distribution with normalized counts and dispersion. The proportion of the gene counts in the sample to the concentration of cDNA was scaled by a normalization factor using the median-of-ratios method. The variability between replicates is modeled by the dispersion parameter using empirical Bayes shrinkage estimation. For each gene, we fit a generalized linear model to get the overall expression strength of the gene and the log 2-fold change between CV and MF groups. For significance testing, differential gene expression is determined by the Wald test. The resulting p-values were corrected for multiple comparisons using the Benjamini-Hochberg FDR method^69^. Genes with an adjusted P-value < 0.05 and |log_2_FoldChange | > 1 were assigned as differentially expressed.

To get a better annotation of the honeybee reference genome, we re-annotate it using eggNOG-mapper v5.0^70^. 6,269 out of 12,375 honeybee genes were successfully assigned to a KO entry with the ‘diamond’ mode, and the hierarchy information of the KEGG metabolic pathway was extracted. Functional analysis of differentially expressed genes was performed based on KEGG Orthologue (KO) markers. The percentages of KO markers belong to each category (KEGG Class at level 3) out of total CV- and MF-enriched KO markers were designated as a comparison parameter. The significance level was calculated by Fisher’s exact test using clusterProfiler v3.10.1^71^.

### Brain proteome analysis

The proteome analysis was performed as described by Meng et al.^72^. Briefly, three biological replicates per treatment group were analyzed for each group of bees. 20 dissected honeybee brains were pestle ground, sonicated, and cooled on ice for 30 min in a lysis buffer (8 M urea, 2 M thiourea, 4% 3-((3-cholamidopropyl) dimethylammonio)-1-propanesulfonate acid (CHAPS), 20 mM tris-base, 30 mM dithiothreitol (DDT)). The homogenate was centrifuged at 12,000 g and 4 °C for 15 min, followed by supernatant recovery. Then 4 volumes of ice-cold acetone were added for 30 min to precipitate protein. The protein pellets were collected after centrifugation (8,000 g, 4 °C for 15 min), dried at room temperature, and dissolved in 40 mM NH_4_HCO_3_. To prevent reformation of disulfide bonds, the dissolved protein samples were incubated with 100 mM of DDT (DDT/protein (V: V = 1:10)) for 1 h and then alkylated with 50 mM of iodoacetamide (IAA) (DDT/IAA (V: V = 1:5)) for 1 h in the dark. Finally, the resultant protein was digested with trypsin (enzyme: protein (W: W = 1:50)) at 37 °C for 14 h. After digestion, the enzymatic reaction was stopped by adding 1 μL of formic acid into the mixture. The digested peptides were centrifuged at 13,000 g and 4 °C for 10 min. The supernatant was recovered and extracted using a SpeedVac system (RVC 2-18, Marin Christ; Osterod, Germany) for subsequent LC-MS/MS analysis.

Peptides were measured by the EASY-nLC 1000 liquid chromatograph (Thermo Fisher Scientific, Waltham, MA, USA) on a Q Exactive HF mass spectrometer (Thermo Fisher Scientific). Peptides were separated on an analytical column packed with 2 μm Aqua C18 beads (15 cm long, 50 μm inner diameter, Thermo Fisher Scientific) at a flow rate of 350 nL/min, using a 120-min gradient (2% (vol/vol) to 10% (vol/vol) acetonitrile with 0.1% (vol/vol) formic acid). The Q Exactive was operated in the data-dependent mode with the following settings: 70000 resolution, 350–1,600 *m/z* full scan, Top 20, and a 2 *m/z* isolation window. Identification and label-free quantification of peptides were done with PEAKS Studio X + (Bioinformatics Solutions Inc.; Waterloo, ON, Canada) against the sequence database (21,780 protein sequences of *Apis mellifera*), coupled with a common repository of adventitious proteins database (cRAP, https://www.thegpm.org/dsotw_2012.html). The search parameters were: parent ion tolerance, 15 ppm; fragment tolerance, 0.05 Da; enzyme, trypsin; maximum missed cleavages, 3; fixed modification, carbamidomethyl (C, + 57.02 Da); and variable modification, oxidation (M, + 15.99 Da). A protein was confidently identified only if it contained at least one unique peptide with at least two spectra, applying a threshold of false discovery rate (FDR) ≤ 1.0% by a fusion-decoy database searching strategy^73^. Proteins significantly differential between groups were identified using ANOVA (*p*-value < 0.05 and a fold change of ≥1.5).

The functional gene ontology (GO) term and pathway were assessed using ClueGOv2.5.5, Cytoscape plug-in software (http://www.ici.upmc.fr/cluego/). The analysis was performed by comparing an input data set of identified proteins to all functionally annotated GO categories in the entire genome of *Apis mellifera* from UniProt. The significantly enriched GO terms in cellular component (CC), molecular function (MF), biological processes (BPs), and pathways were reported using a two-sided hypergeometric test, and only a *p*-value ≤ 0.05 was considered. Then, the Bonferroni step-down was used to correct the p-value to control FDR. Functional grouping of the terms was based on the GO hierarchy. The tree-level was ranged from 3 to 8, and the kappa score level was 0.4.

### Quasi-Targeted metabolomics analysis

Hemolymph and gut homogenate metabolites were determined by quasi-targeted metabolomics by HPLC-MS/MS. Gut samples (100 mg) were individually grounded with liquid nitrogen, and the homogenate was resuspended with prechilled 500 μl 80% methanol and 0.1% formic acid by well vortexing. 50 μl of hemolymph samples were mixed with 400 μl prechilled methanol by vortexing. All samples were incubated on ice for 5 min and then centrifuged at 15,000 × *g*, at 4 °C for 10 min. The supernatant was diluted to a final concentration containing 53% methanol by LC-MS grade water. The samples were then transferred to a fresh vial and centrifuged at 15,000 × *g*, 4 °C for 20 min. Finally, the supernatant was injected into the LC-MS/MS system, and the analyses were performed using an ExionLC AD system (SCIEX) coupled with a QTRAP 6500+ mass spectrometer (SCIEX). Samples were injected onto a BEH C8 Column (100 mm × 2.1 mm × 1.9 μm) using a 30-min linear gradient at a flow rate of 0.35 ml/min for the positive polarity mode. Eluent A was 0.1% formic acid-water, and eluent B was 0.1% formic acid-acetonitrile. The solvent gradient was set as follows: 5% B, 1 min; 5–100% B, 24.0 min; 100% B, 28.0 min;100–5% B, 28.1 min;5% B, 30 min. QTRAP 6500+ mass spectrometer was operated in positive polarity mode with curtain gas of 35 psi, collision gas of medium, ion spray voltage of 5500 V, the temperature of 500 °C, ion source gas of 1:55, and ion source gas of 2:55. For negative ion mode, samples were injected onto aHSS T3 Column (100 mm × 2.1 mm) using a 25-min linear gradient at a flow rate of 0.35 ml/min. The solvent gradient was set as follows: 2% B, 1 min; 2%–100% B, 18.0 min; 100% B, 22.0 min; 100%–5% B, 22.1 min; 5% B, 25 min. QTRAP 6500+ mass spectrometer was operated in negative polarity mode with curtain gas of 35 psi, collision gas of medium, ion spray voltage of -4500V, the temperature of 500 °C, ion source gas of 1:55, and ion source gas of 2:55.

Detection of the experimental samples using MRM was based on Novogene in-house database. Q3 (daughter) was used for the quantification. Q1 (parent ion), Q3, retention time, declustering potential, and collision energy were used for metabolite identification. Data files generated by HPLC-MS/MS were processed with SCIEX OS (version 1.4) to integrate and correct the peaks. A total of 326 compounds were identified in the hemolymph and gut samples. Metabolites identified in the gut of MF and CV bees were obtained from previous study^9^. Metabolomics data analysis was then performed using MetaboAnalyst 4.0^74^.

### Statistical analysis

Comparison of the learning and memory results was tested by Chi-squared test. Comparisons of normalized and raw metabolite data, and the expression level of TDO and AhR genes of different groups were made by Mann–Whitney *u* test. The exact value of n representing the number of groups in the experiments described was indicated in the figure legends. Any additional biological replicates are described within the Methods and the Results.

### Reporting Summary

Further information on research design is available in the Nature Research Reporting Summary linked to this article.

## Supplementary information

Supplementary Information

Description of Additional Supplementary Files

Dataset S1

Dataset S2

Dataset S3

Dataset S4

Dataset S5

Supplementary Movie 1

Reporting Summary

## Data Availability

The raw data for outdoor honeybee gut microbiome shotgun sequencing has been deposited under BioProject PRJNA670603. The accession numbers for the RNA sequencing data are PRJNA743412. The proteomic data has been deposited to the Proteome Xchange Consortium with the dataset identifier PXD022304. All data supporting the findings of this study are available in the manuscript or supplementary information. Source data are provided with this paper.

## Source data

Source Data

## Code availability

The list of analysis software and all scripts generated for analysis have been deposited on GitHub at: https://github.com/ZijingZhang93/bee_BGA.git.
