# Supplementary material for: Honeybee gut Lactobacillus modulates host learning and memory behaviors via regulating tryptophan metabolism
Source: Nat Commun. 2022 Apr 19;13:2037. doi: 10.1038/s41467-022-29760-0 (PMC9018956; doi:10.1038/s41467-022-29760-0)
Supplement: Supplementary file 1 — Supplementary Information [file 41467_2022_29760_MOESM1_ESM.pdf]

Supplementary Information: Honeybee gut *Lactobacillus* modulates host learning and memory behaviors via regulating tryptophan metabolism

H. Zheng et al.

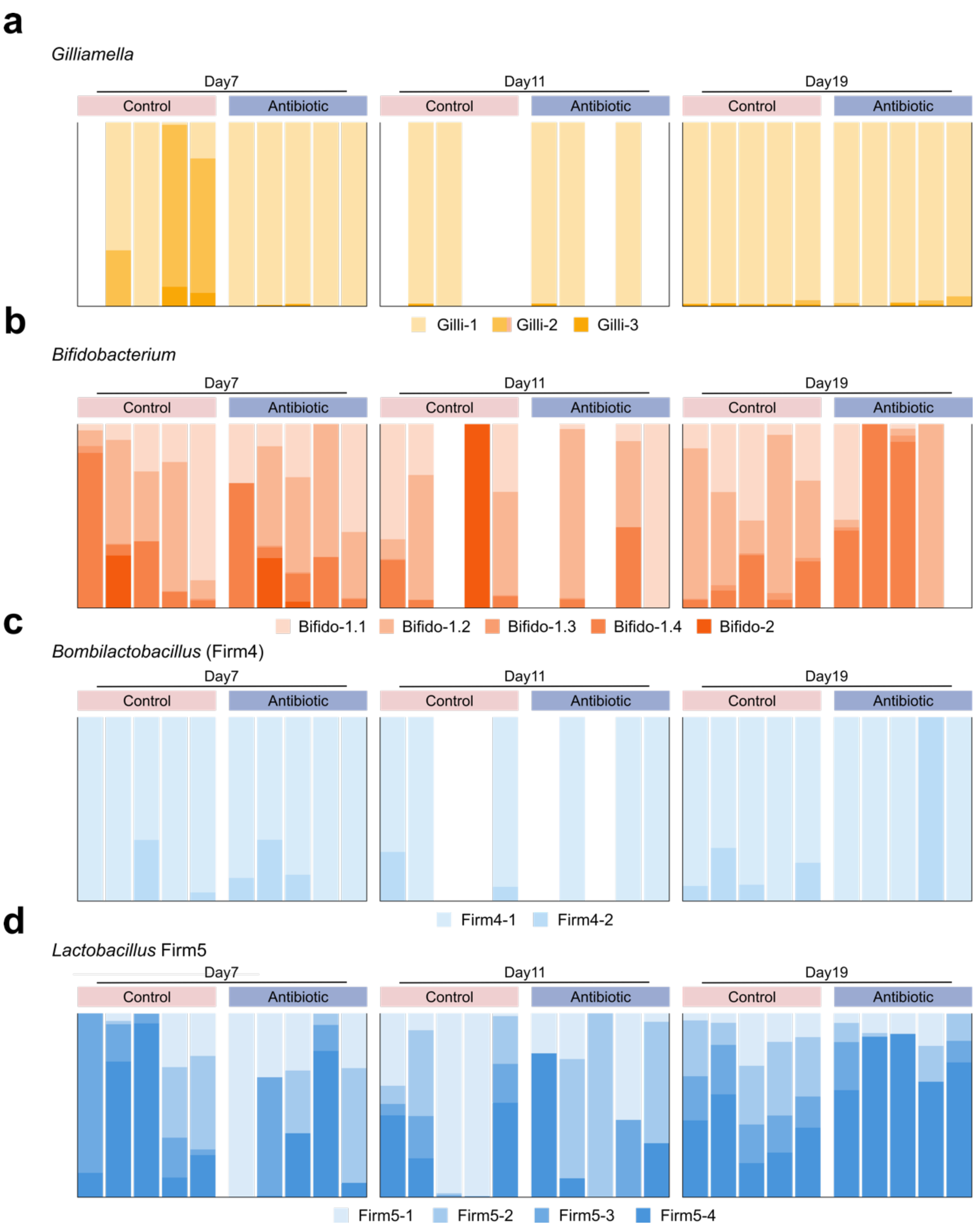

Supplementary Fig. 1. Antibiotic treatment affects the gut microbiota of honeybees. **a**-**d** Compositions at SDP-level for four core bee gut members: (**a**) *Gilliamella*, (**b**) *Bifidobacterium*, (**c**) *Bombilactobacillus* (Firm4), and (**d**) *Lactobacillus* Firm5. Source data are provided as a Source Data file.

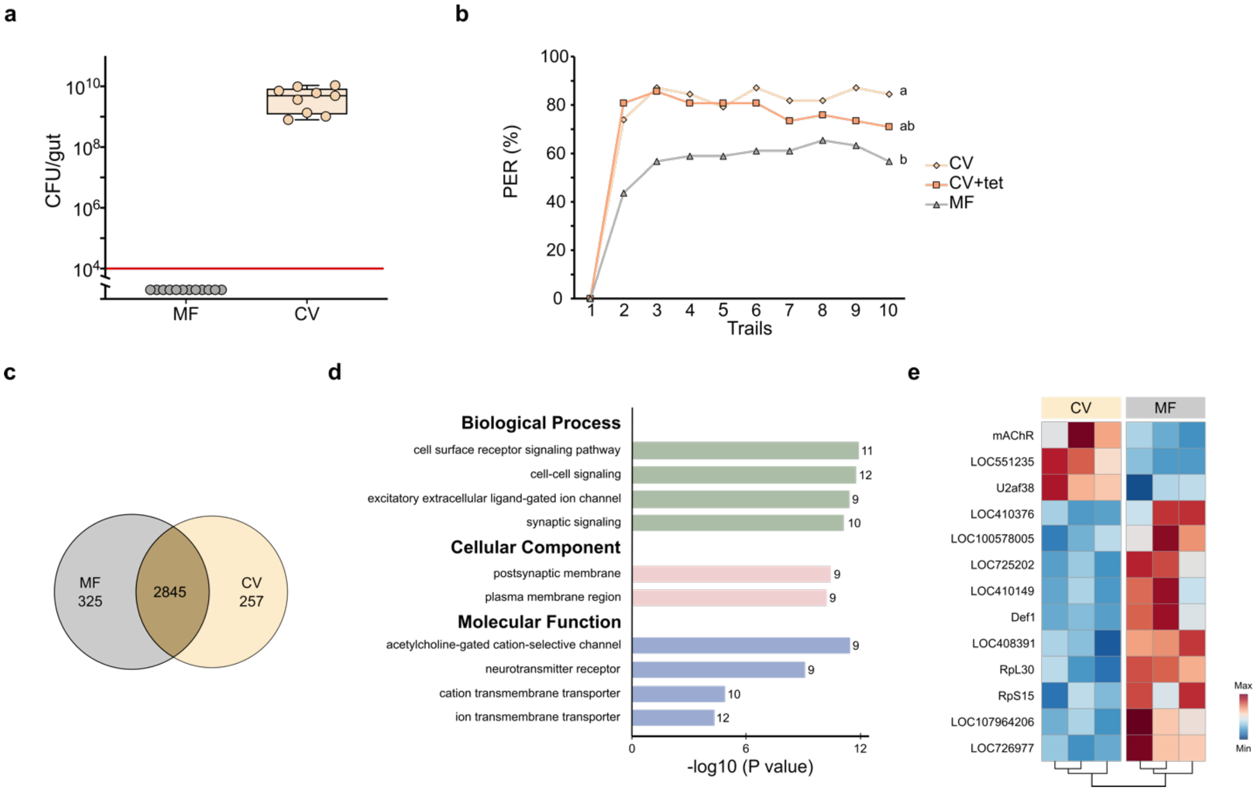

Supplementary Fig. 2. Gut microbiota impacts learning performance and the proteomic profiling in the honeybee brain. a Boxplots of the total CFU per gut estimated by bacteria culture for MF bees (n = 12), or by qPCR for the CV group (n = 9). Error bars represent min and max. **b** Acquisition curves (% of PER to the nonanol odor) of bees in CV (n = 38), CV+tet (n = 41), and MF groups (n = 46) during 10 training trials. Different letters (a, b, c) stand for statistical differences between groups (two-sided Fisher's exact test: p = 0.0088 between MF and CV groups; p = 0.1862 between CV+tet and CV groups). **c** Venn diagrams indicating the numbers of shared and unique proteins identified in the brains of CV and MF bees. **d** The most significantly enriched GO terms of identified proteins unique to the CV group (p < 0.0001, one-way ANOVA test). The number of genes involved in each GO term is displayed next to the bars. **e** Heatmap of differentially expressed proteins detected in the brains of CV and MF bees. Source data are provided as a Source Data file.

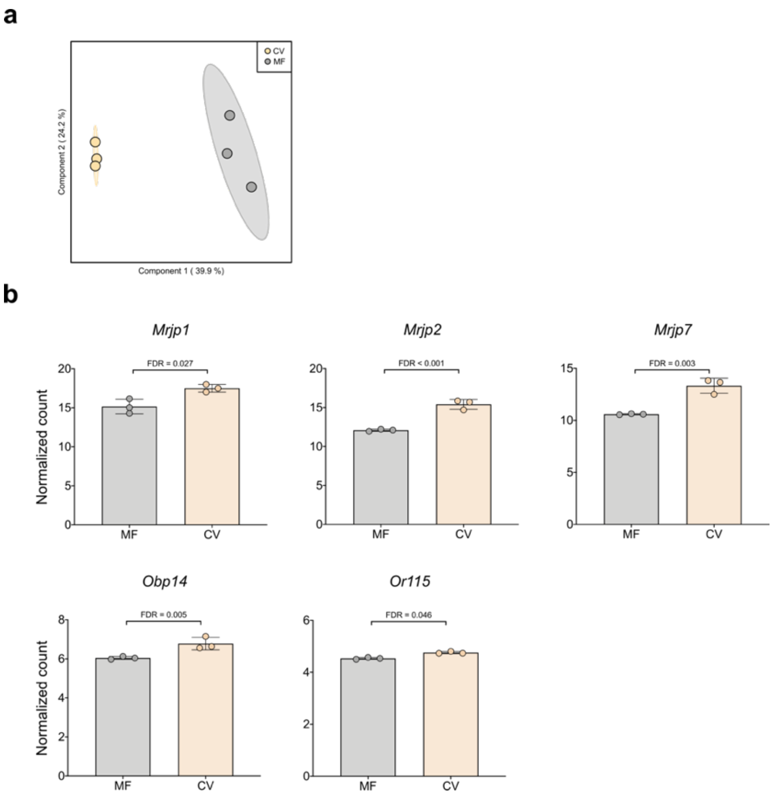

Supplementary Fig. 3. Gut microbiota impacts transcriptomic profile in the honeybee brain. a Sparse PLS-DA based on normalized gene expression in the brain of MF and CV bees. **b** Relative expression levels of differentially expressed genes in the brains of MF and CV groups (*n* = 3 bees for both groups). Differences between CV bees and the MF group were tested by Wald test with Benjamini-Hochberg correction. Data are presented as mean ± SD. Source data are provided as a Source Data file.

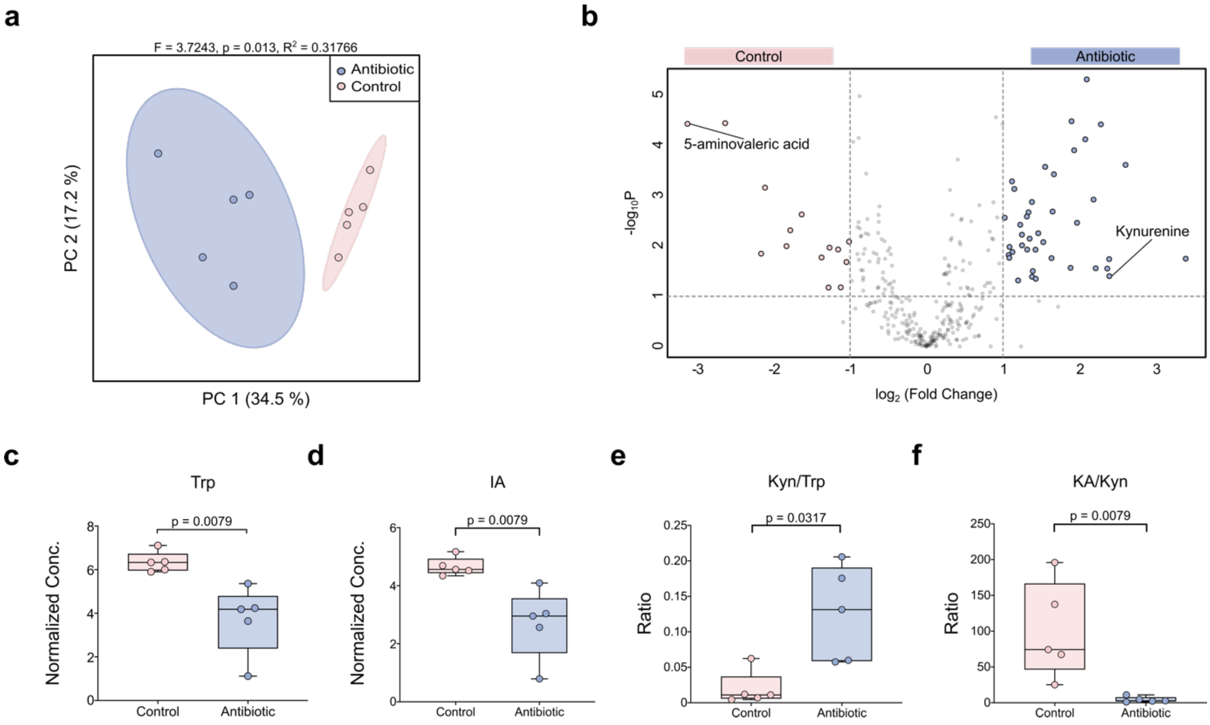

Supplementary Fig. 4. Antibiotic treatment affects gut Trp metabolism under field conditions. a Principal coordinate analysis based on all metabolites detected in guts of control and antibiotic-treated bees. Group differences were tested by permutational multivariate ANOVA (PERMANOVA). **b** Volcano plot showing the differentially regulated metabolites. Metabolites significantly enriched in control bees are shown in pink, and those enriched in antibiotic-treated bees are in blue. **c-f** Boxplots of (**c**) the normalized concentration of Trp and (**d**) IA, (**e**) the Kyn/Trp ratio, and (**f**) the KA/Kyn ratio in the guts of control and antibiotic-treated bees (n = 5 bees for both groups). Group differences were tested by two-sided Mann-Whitney u test. Error bars represent min and max. Source data are provided as a Source Data file.

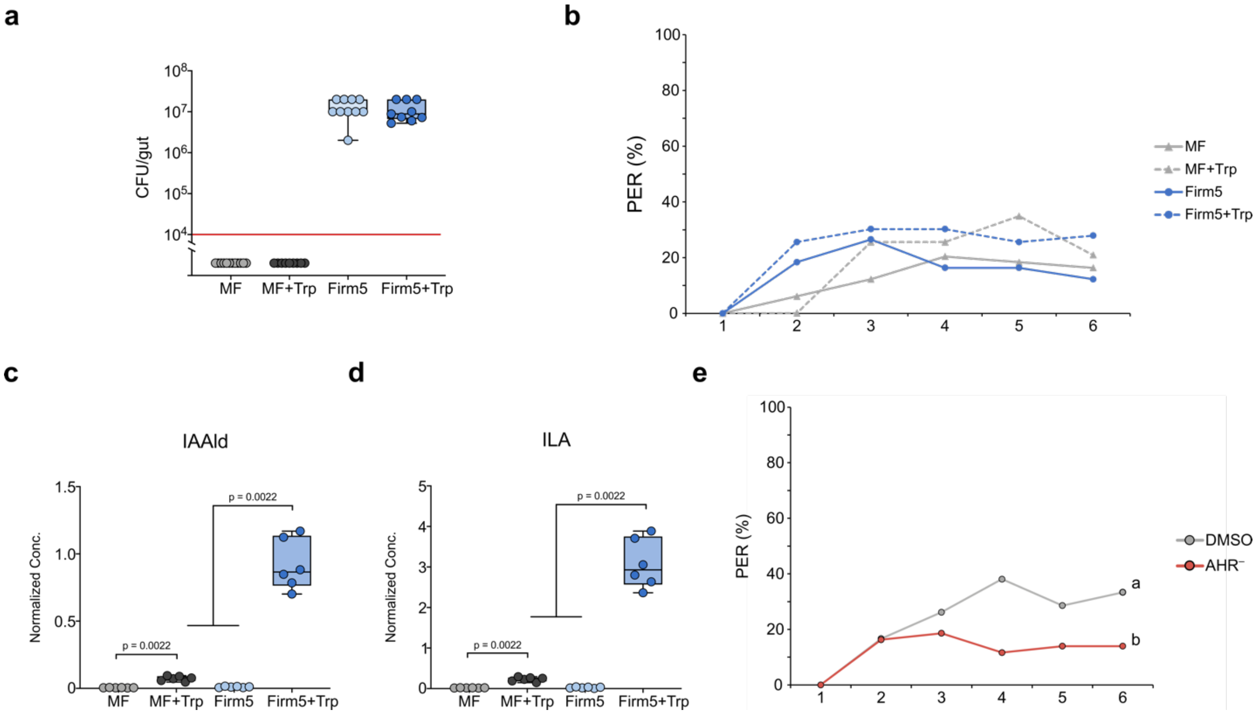

Supplementary Fig. 5. ***Lactobacillus apis*** **modulates honeybee learning performance via the activation of AhR. a** Boxplots of the total CFU per gut estimated by bacteria culture for MF (n = 12), MF+Trp (n = 9), Firm5 (n = 10), and Firm5+Trp (n = 9) bees. Error bars represent min and max. **b** Acquisition curves (% of PER to the nonanol odor) of bees in MF (n = 49, grey triangles), MF+Trp (n = 43, grey triangles with dotted line), Firm5 (n = 49, blue circles), and Firm5+Trp groups (n = 43, blue circles with dotted line) during 6 training trials. Different letters (a and b) stand for statistical differences between groups (Chi-square test, p = 0.2617). **c-d** Boxplots of the normalized concentration of (**c**) indole-3-acetaldehyde (IAAld) and (**d**) indole-3-lactic acid (ILA) in the gut of MF, MF+Trp, Firm5, and Firm5+Trp bees (n = 5 bees for all groups). Group differences were tested by two-sided Mann-Whitney *u* test. Error bars represent min and max. **e** Acquisition curves (% of PER to the nonanol odor) of bees in DMSO (n = 42, grey circles) and AHR^{-} (n = 43, red circles) during 6 training trials. Different letters (a and b) stand for statistical differences between groups (two-sided Fisher's exact test: p = 0.0431). Source data are provided as a Source Data file.
