# Supplementary material for: Honeybee gut Lactobacillus modulates host learning and memory behaviors via regulating tryptophan metabolism
Source: Nat Commun. 2022 Apr 19;13:2037. doi: 10.1038/s41467-022-29760-0 (PMC9018956; doi:10.1038/s41467-022-29760-0)
Supplement: Supplementary file 2 — Description of Additional Supplementary Files [file 41467_2022_29760_MOESM2_ESM.docx]

File Name: Supplementary Data 1

Description: The list of genomes of bacterial isolates in the database for MIDAS profiling.

File Name: Supplementary Data 2

Description: Identification and biological function analysis of proteins expressed in brains of microbiota-free and conventional bees.

File Name: Supplementary Data 3

Description: Normalized gene expression levels in brains of microbiota-free and conventional bees.

File Name: Supplementary Data 4

Description: Raw data of all metabolite abundance in the hemolymph of microbiota-free and conventional bees in laboratory conditions.

File Name: Supplementary Data 5

Description: Raw data of all metabolite abundance in the gut of antibiotic-treated, and control bees under field conditions.

File Name: Supplementary Movie 1

Description: Olfactory learning and memory test.
