# Supplementary material for: Honeybee gut Lactobacillus modulates host learning and memory behaviors via regulating tryptophan metabolism
Source: Nat Commun. 2022 Apr 19;13:2037. doi: 10.1038/s41467-022-29760-0 (PMC9018956; doi:10.1038/s41467-022-29760-0)
Supplement: Supplementary file 9 — Reporting Summary [file 41467_2022_29760_MOESM9_ESM.pdf]

nature research

Corresponding author(s): Hao Zheng

Last updated by author(s): Jan 17, 2022

Reporting Summary

Nature Research wishes to improve the reproducibility of the work that we publish. This form provides structure for consistency and transparency in reporting. For further information on Nature Research policies, see our Editorial Policies and the Editorial Policy Checklist.

Statistics

For all statistical analyses, confirm that the following items are present in the figure legend, table legend, main text, or Methods section.

n/a	Confirmed	
☐	☒	The exact sample size (n) for each experimental group/condition, given as a discrete number and unit of measurement
☐	☒	A statement on whether measurements were taken from distinct samples or whether the same sample was measured repeatedly
☐	☒	The statistical test(s) used AND whether they are one- or two-sided<br>*Only common tests should be described solely by name; describe more complex techniques in the Methods section.*
☐	☒	A description of all covariates tested
☐	☒	A description of any assumptions or corrections, such as tests of normality and adjustment for multiple comparisons
☐	☒	A full description of the statistical parameters including central tendency (e.g. means) or other basic estimates (e.g. regression coefficient) AND variation (e.g. standard deviation) or associated estimates of uncertainty (e.g. confidence intervals)
☐	☒	For null hypothesis testing, the test statistic (e.g. F, t, r) with confidence intervals, effect sizes, degrees of freedom and P value noted<br>*Give P values as exact values whenever suitable.*
☒	☐	For Bayesian analysis, information on the choice of priors and Markov chain Monte Carlo settings
☒	☐	For hierarchical and complex designs, identification of the appropriate level for tests and full reporting of outcomes
☒	☐	Estimates of effect sizes (e.g. Cohen's d, Pearson's r), indicating how they were calculated

Our web collection on statistics for biologists contains articles on many of the points above.

Software and code

Policy information about availability of computer code

Data collection	For metagenomic and transcriptomic data collection, Illumina NovaSeq 6000 platform was used. For metabolomic data collection, ExionLC AD system (SCIEX) and QTRAP 6500+ mass (SCIEX) were used. For proteomic data collection, EASY-nLC 1000 (Thermo) and Q-Exactive HF (Thermo Fisher) were used. For quantitative real-time PCR data collection, QuantStudio Design & Analysis Software (Version 1.5.1) was used.
Data analysis	All scripts generated for analysis have been deposited on GitHub at https://github.com/ZijingZhang93/bee_BGA.git. Statistical analysis were performed with R (version 3.2.0).

For manuscripts utilizing custom algorithms or software that are central to the research but not yet described in published literature, software must be made available to editors and reviewers. We strongly encourage code deposition in a community repository (e.g. GitHub). See the Nature Research guidelines for submitting code & software for further information.

Data

Policy information about availability of data

All manuscripts must include a data availability statement. This statement should provide the following information, where applicable:

- Accession codes, unique identifiers, or web links for publicly available datasets
- A list of figures that have associated raw data
- A description of any restrictions on data availability

Raw sequencing reads are freely available on the Short Read Archive (SRA) under BioProject ID PRJNA670603 (http://www.ncbi.nlm.nih.gov/bioproject/670603), PRJNA668910 (http://www.ncbi.nlm.nih.gov/bioproject/668910), and PRJNA670620 (http://www.ncbi.nlm.nih.gov/bioproject/670620). The proteomic data has been deposited to the Proteome Xchange Consortium with the dataset identifier PXD022304 (http://proteomecentral.proteomexchange.org/cgi/GetDataset?ID=PXD022304). All data supporting the findings of this study are available in the manuscript or the supplementary information. Source data are provided with this paper.

Field-specific reporting

Please select the one below that is the best fit for your research. If you are not sure, read the appropriate sections before making your selection.

- [x] Life sciences
- [ ] Behavioural & social sciences
- [ ] Ecological, evolutionary & environmental sciences

For a reference copy of the document with all sections, see nature.com/documents/nr-reporting-summary-flat.pdf

Life sciences study design

All studies must disclose on these points even when the disclosure is negative.

Sample size	Sample sizes were chosen based on those used in similar studies to which we expected similar effect sizes in our current experiments (Zheng et al., 2017, PNAS; Kešnerová et al., 2017, Plos Biol.; Emery et al., 2017, Mol. Ecol.; Meng et al., 2018, Mol. Cell Proteomics).
Data exclusions	No data were excluded from the analyses.
Replication	Behavioral data were reproduced by independent experimenters.<br>For all experiments, at least three biological replicates were used, as noted in the manuscript, to yield reproducible results. All attempts at replication were successful.
Randomization	Bees were randomly assigned to different treatment groups.
Blinding	All analysis were performed blind.

Reporting for specific materials, systems and methods

We require information from authors about some types of materials, experimental systems and methods used in many studies. Here, indicate whether each material, system or method listed is relevant to your study. If you are not sure if a list item applies to your research, read the appropriate section before selecting a response.

Materials & experimental systems

n/a	Involved in the study	
x		Antibodies
x		Eukaryotic cell lines
x		Palaeontology and archaeology
	x	Animals and other organisms
x		Human research participants
x		Clinical data
x		Dual use research of concern

Methods

n/a	Involved in the study	
x		ChIP-seq
x		Flow cytometry
x		MRI-based neuroimaging

Animals and other organisms

Policy information about studies involving animals; ARRIVE guidelines recommended for reporting animal research

Laboratory animals	Worker honeybees (Apis mellifera) were used in this study. All bees are 7-day-old at time of collection.
Wild animals	The study did not involve wild animals.
Field-collected samples	For all experiments, honeybee (Apis mellifera) were collected from the apiary of China Agricultural University, Beijing, China during the Summer and Autumn 2019. Brood frames were collected from a single hives and transfered to the lab. For laboratory experiment, late-stage pupae were removed manually from brood frames and placed in sterile plastic bins. Then the pupae emerged in an incubator at 35°C, with humidity of 50%. For field experiment, frames were then kept in the laboratory incubating at 35°C and 50% relative humidity for two days. Then emerged bees were labeled and introduced to empty hives together with a newly mated laying queen under field condition.
Ethics oversight	No ethical approval was required in this case.

Note that full information on the approval of the study protocol must also be provided in the manuscript.
